# A Systematic Review of Individual Motivational Factors in Orthodontic Treatment: Facial Attractiveness as the Main Motivational Factor in Orthodontic Treatment

**DOI:** 10.1155/2014/938274

**Published:** 2014-05-20

**Authors:** Lusine Samsonyanová, Zdenek Broukal

**Affiliations:** Institute of Clinical and Experimental Dental Medicine, 1st Faculty of Medicine, Charles University in Prague, Karlovo Náměstí 32, 121 01 Prague, Czech Republic

## Abstract

*Introduction.* Physical, mental, and social consequences of malocclusion may impact the quality of life. The aim of this review is to describe main factors motivating parents for orthodontic treatment for their children. *Methods.* A systematic review study design was used to identify articles analyzing different motivational factors in orthodontic treatment appearing in Medline database, EMBASE, and Google Scholar. The search terms used were teasing, motivating factors, orthodontics, malocclusion, quality of life, smile attractiveness, and perception of malocclusion. Papers selected up to May 2013 included retrospective and prospective longitudinal studies, randomized control trials, cross-sectional studies, reviews, and meta-analyses. *Results.* 13 articles included in this review identified aesthetics as the main motivational factor in orthodontic treatment. Children mention teeth crowding, large overbite, missing teeth, and largest maxillary anterior irregularities also as motivational factors. Parents want their children to look nice and worry of being accused of neglecting parental duties. *Conclusions.* Dissatisfaction with one's appearance, dentist recommendation, interest and worries of parents, and the impact of peers who wear braces rank among the main motivation factors of seeking orthodontic treatment. Understanding these factors allows better planning of resources and better assessment of the requirements and priorities of treatment.

## 1. Introduction

Physical attractiveness affects human life in various ways and to a significant extent. It has been proven that the face is a slightly stronger indicator of overall attractiveness than the body [1]. Attractive people are regarded as friendly, intelligent, interesting, more social, and much more positive personalities [2–4]. Irregularities in the position of the teeth and jaws have a significant impact on the attractiveness and aesthetics of the smile and on quality of life. These irregularities can disrupt social interaction, interpersonal relationships, and mental wellbeing and may lead to a feeling of inferiority [5].

Most orthodontic patients are children and adolescents [6, 7]. It is assumed that an irregular set of teeth and less aesthetic face can negatively affect a child. The child is then the target of jibes and is given nicknames and so forth [3, 8]. Most parents seek specialised orthodontic care for their children to improve their overall appearance. It is important to identify factors which directly motivate parents to bring their child in for orthodontic examination and as the case maybe orthodontic treatment.

The aim of this paper is to give a systematic review of motivational factors for orthodontic treatment in children. The authors think that understanding the factors which contribute towards seeking out orthodontic treatment allow for better planning of resources and better assessment of the requirements and priorities of treatment. The secondary aim of this paper is also to give an overview about the facial attractiveness and social stereotyping, respectively, on the impact of facial attractiveness on quality of life.

## 2. Materials and Methods

A comprehensive electronic database search to identify relevant publications was conducted, and the reference lists in relevant articles were searched manually for additional literature. We used a systematic review study design. Medline database, EMBASE, and Google Scholar were searched for articles published. Searching papers included retrospective and prospective longitudinal studies, randomized control trials, and cross-sectional studies to determine individual motivational factors of parents in orthodontic treatment. The last electronic search was concluded in May 2013. We were searching articles published in English.

The search strategy focused on the following terms: “teasing, motivation in orthodontic treatment, malocclusion and quality of life, smile attractiveness, and smile aesthetic perception.”

**Figure 1:**
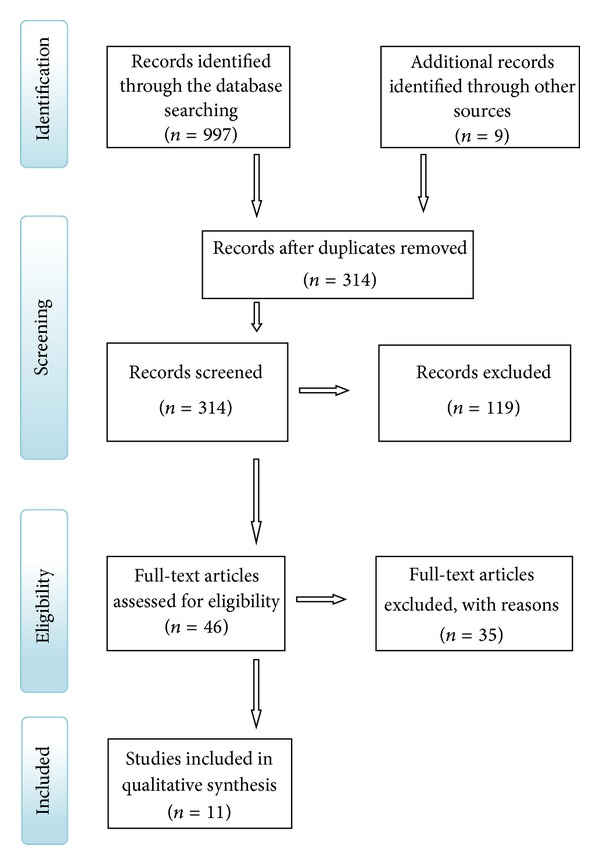
Methodology followed in the article selection process (adapted from Moher et al. [22]).

The initial search revealed 997 articles that were found using the searching strategy and only the titles related to orthodontic treatment were selected. The number of articles reviewed in each phase to perform this systematic review is presented in the PRISMA flow diagram (Figure 1). The second stage of the search protocol was to retrieve the reference lists of the selected articles, which yielded 9 additional articles of interest. After excluding 683 duplicates, 314 articles remained for review. In the first phase selection, the screening of the articles by reading titles and abstracts was proceeding. Articles that were not eligible because of irrelevant aims and were not directly related to this systematic review were excluded; thus, 222 articles remained for further reading. 46 articles were assessed for eligibility.

After screening, all the 11 articles were selected for qualitative synthesis.

## 3. Results

11 articles were selected for systematic review (Table 1). Main reason for children to undergo the orthodontic treatment was aesthetics. Crowding of the teeth and large overbite were reported as main motivational factors in study of Tung and Kiyak [3]. In study of Tessarollo et al. [9], dissatisfaction with dental appearance in children and adolescents was missing teeth and when largest maxillary anterior irregularity is present. Children also report that orthodontic treatment can improve their quality of life that it can be easier to get a job thanks to orthodontic treatment, and that it is easier to find a romantic partner [10]. In the same study, children report discrimination when smiling on the part of schoolmates. From 77% responders in one article who reported teasing, only 4.7% of them reported teasing and nicknames because of teeth (equal in boys and girls). The author conclude that dental appearance may not be a significant contributor to nicknames [6]. In the other article [11], 44% of parents report teasing of their children because of teeth. Parents of children with overjet ≥7 mm are 5.5 times as likely to report that their child had been teased when compared to parents of children with lesser overjet. The same article reports, as other reasons for interest in orthodontic treatment, 85% appearance of teeth, 46% facial appearance, 16% speech, and 73% dentist's advice.

Parents also report, as the main motivational factor, aesthetics, precisely irregular positioning of the teeth. Parents want their children to look nice. Another reason is the fear of being accused that they neglected their parental duties [3, 12]. They consider anterior crowding ≥2 mm as the reason for orthodontic treatment of their children [10]. Parents consider that orthodontic treatment would enhance oral health and enhance self-esteem [13]. Kilpeläinen et al. [11] report that 85% of parents in their study, as a motivational factor, consider appearance of teeth; 46% of them report facial appearance, and 16% report speech. It is interesting in this study that 73% of respondents report that the dentist's advice was a motivational factor for their children treatment.

## 4. Literature Review and Discussion

### 4.1. Attractiveness of the Face

The main factor determining attractiveness is a person's face. Better looking people are regarded as friendly, more intelligent, much more interesting, and much more socially competent [2, 14]. The reason why people seek orthodontic consultation as a result of this is their wish to improve their appearance. The ideal of beauty is subject to certain fashion trends [2]. The orthodontist tries to fulfil the patient's expectations to straighten crooked teeth by following specific standard procedures and rules. Nevertheless, it is stated in the literature [5] that some standards do not correspond to that which the layman perceives as beauty.

**Figure 2:**
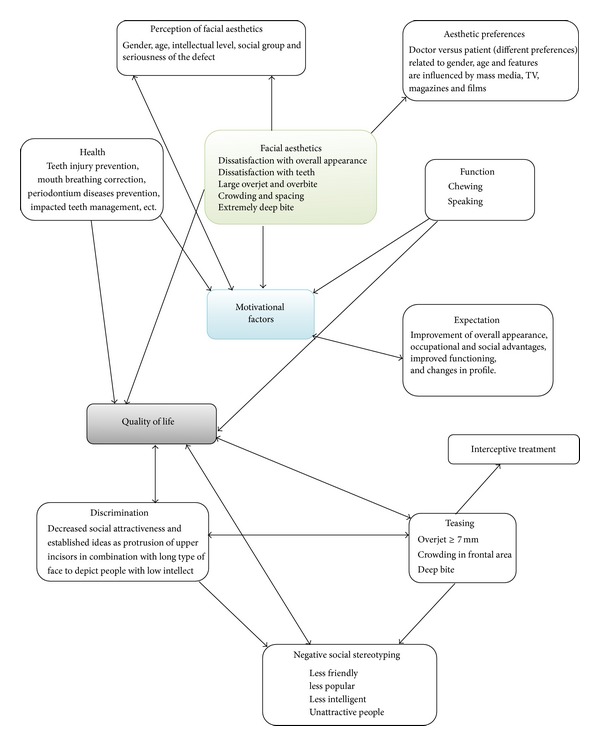
Impact of facial aesthetics on quality of life.

Attractiveness is judged on the basis of social standards. In addition to this, the literature also points to the fact that people have a natural ability to distinguish between the beautiful and the ugly. Numerous studies performed by Professor Langlois et al. [15] show that even children pay greater attention to people with a more attractive face than people of less attractive appearance. The connection between facial aesthetics, quality of life, and motivational factors for treatment is explained in Figure 2.

### 4.2. Symmetry and Facial Attractiveness

Many authors are convinced that a perfectly symmetrical face has a definite impact of the attractiveness of the face. In his study, Cellerino [16] came to the conclusion that symmetry may contribute towards attractiveness but that it is not a decisive factor for the attractiveness of the face. Other authors do not regard facial symmetry as important but claim that asymmetrical faces are perceived as less attractive [16].

### 4.3. Public Taste in Facial Aesthetics

The opinions of doctors in the concept of ideas about facial aesthetics differ [17], and that what appears aesthetic to some is not liked by others. The same study confirms the opinion that, in the American population, white features are considered to be more attractive than Negroid (African) features. The study also claims that it is highly likely that the mass media have a great influence on unifying people's taste. Television, films, newspapers, and magazines provide daily indoctrination regarding certain facial stereotypes. The orthodontist is subject to cultural preconceptions just like other people. Nevertheless, the interest of the orthodontist in facial aesthetics is more academic than emotional.

### 4.4. Facial Attractiveness and “Body Image”

Current findings claim that irregularities in the position of the teeth and jaws have physical, mental, and social consequences which have an impact on the quality of life [18]. One example of this is a study which states that class II malocclusion can lead to psychosocial problems such as mockery, negative stereotyping, and low self-confidence [19]. Interceptive treatment is recommended here to avoid the creation of low self-confidence. The way in which individuals perceive their body plays an important role in the feeling of safety and self-confidence. It is generally acknowledged that a strong correlation exists between physical appearance, especially facial aesthetics, and social attractiveness [19]. It may logically seem that improvement of facial aesthetics in the individual will have a positive impact on “body image.” But this claim is controversial. Despite the fact that improvement of facial aesthetics is the primary reason for seeking orthodontic treatment [20], there is little evidence to support the connection between lacks of bite defects and measurably greater self-confidence [19]. Social stereotyping, based on facial aesthetics, disproportionately affects adolescents and young adults. Furthermore, it could be the main factor in adapting oneself to life.

### 4.5. Facial Attractiveness and Teasing

Children who are regarded as more attractive are more accepted by their peers and those around them regard them as more intelligent. These individuals are more desirable as friends [3]. It has been proven that irregularities in the position of the teeth and jaws are a cause of teasing and harassment among children and that they relate to decreased social attractiveness [20]. Adolescents and adults with abnormalities in the position of their teeth and jaws may come up against discrimination in various environments [20]. The existence of these established ideas may be found, for example, in animated films: the creators of animated stories typically use protruding upper incisors and a long type of face to depict people of low intellect and caricatures with a small upper jaw and prominent chin to depict the traits of a witch [20].

Children of young school age are able to distinguish regular, nice looking teeth from irregular teeth. They are able to recognise crowding, gaps between the teeth, and the generally irregular position of the teeth [21]. Even partial alignment of teeth in sensitive children can be of psychological importance [11]. Other reasons for teasing are crowding of the teeth in the frontal area of the teeth and deep bite.

There were no enough articles to study the common motivational factors in orthodontic treatment. And authors consider that there is a need to determine individual motivational factors for orthodontic treatment from the point of view of the aesthetics, function, and health.

Questionnaire-designed randomized studies about all known motivational factors for orthodontic treatment are still required based on more patiens, devided in different age groups and their parents.

These are possible motivational factors to be included in the future questionnaire.

They include teasing, self-esteem, better life opportunities, more friends, career opportunities, finding a better job, overall smile attractiveness, overjet, spacing, crowded upper teeth, crowded lower teeth, gummy smile, oral habits, clenching or bruxism, mouth breathing, impossible to close mouth, shape of teeth, color of teeth, diastema, missing teeth, problems with biting or chewing, improve dental health, dentist recommendation, and others, individually specified by patient.

## 5. Conclusion

Dissatisfaction with one's appearance, recommendation from a dentist, interest and worries on the part of the parents about neglecting their child's teeth, and the impact of peers who wear braces rank among the main factors which contribute towards seeking out orthodontic treatment. Gender, age, intellectual level, social group, seriousness of the defect, and perception of one's own facial aesthetics also relate to the desire to undergo orthodontic treatment or to provide this to one's children. The influence of these factors depends on the social and cultural characteristics of the population subgroup. Understanding the factors which contribute towards seeking out orthodontic treatment allows for better planning of resources and better assessment of the requirements and priorities of treatment.

The significance of the dentist in recommendation of orthodontic care is important because it is precisely the dentist who has a significant influence on the patient who needs this treatment. At the same time, however, it is also the relationship of the child with their parents which plays an important role in cooperation with the orthodontist. This is why it is important that the factors which influence parental attitude and behaviour are examined.

## Figures and Tables

**Table 1:** Publications related to motivational factors in orthodontic treatment used for systematic review analysis.

Authors (year)	Aim of the study	Subjects	Design of the study	Results and conclusions according to the authors
Wȩdrychowska-Szulc and Syryńska (2010) [12]	To examine patients and parents motivation in orthodontic treatment	674 children who aged 7–18 years and 86 parents who aged 19–42 years	Questionnaire	**Children:** main reason is for aesthetics; less than 5% is influence of their peers **Parents:** 77% seek treatment due to irregular positioning of the teeth, 54% of parents want their children to look nice, and 64% fear of being accused that they neglected their parental duties. Number of patients dissatisfied with the appearance of their teeth increased with age Females demonstrated more concern for appearance than males

Otuyemi and Kolawole (2005) [6]	Perception of orthodontic treatment need. Relationship of the nicknames to dental appearance	506 randomly selected children	Questionnaire	77% responders reported teasing; 4.7% of them reported teasing and nicknames because of teeth (equal in boys and girls). The authors conclude that dental appearance may not be a significant contributor to nicknames

Marques et al. (2009) [10]	To determine factors associated to the desire for orthodontic treatment	403 subjects who aged 14–18 years randomly selected from a population of 182, 291 school children students	Questionnaire	**Children:** 78% expressed a desire to receive orthodontic treatment; 72% of them believed that orthodontic treatment could improve their quality of life; 41% easier to get a job; 27% thought it would be easier to find a romantic partner; 12% discrimination when smiling on the part of schoolmates; 22% status or trend **Parents:** 72% considered it necessary for their child to wear an orthodontic appliance 69% reported that the children were not in treatment due to high costs involved Anterior crowding ≥2 mm

Bennett et al. (1997) [13]	The demand for children's orthodontic care	220 orthodontists and 220 parents	Questionnaire	Orthodontic treatment would enhance oral health and enhance self-esteem

Kilpeläinen et al. (1993) [11]		313 parents were asked to provide answers instead of their children	Questionnaire	44% teasing because of teeth. The reason for interest in orthodontic treatment most frequently selected was as follows: 85% appearance of teeth, 46% facial appearance, 16% speech, and 73% dentist's advice. Parents of children with overjet ≥7 mm are 5.5 times as likely to report that their child had been teased when compared to parents of children with lesser overjet

Tung and Kiyak (1998) [3]	Reasons for orthodontic treatment	75 children and their parents	Questionnaire	**Children:** crowding of the teeth (56%), large overbite (17.3%) **Parents:** 75% of parents were dissatisfied with the appearance of their children's teeth; 54% of them wanted their children “to look pretty”

Daniels et al. (2009) [23]	Orthodontic treatment motivation of patient and parents	227 patients of 7–16 years old and their parents	Questionnaire	91.6% of the parents and 93.4% of children rated aesthetic concerns as the most important Parents were significantly more motivated for their child to have orthodontic treatment than their children

Pratelli et al. (1998) [7]	Parental perception and attitudes in orthodontic treatment	437 parents of 9-year-old children	Questionnaire	Interest on the part of the parents Parents who had been treated themselves or who desired treatment or regretted not being treated or were dissatisfied with their own occlusion perceived orthodontic need in their child

Miner et al. (2007) [24]	The perception of children's profiles by mothers	24 patients and their parents	Computer imaging program	Mothers' perceptions are the primary motivating factors for seeking orthodontic treatment

Tessarollo et al. (2012) [9]	Dissatisfaction with dental appearance	704 adolescents who aged 12-13 years	Questionnaire	Missing teeth Largest maxillary anterior irregularity

Abdullah et al. (2001) [25]	Reasons for seeking orthodontic treatment	110 patients who aged 11–30 years	Questionnaire	65% the desire to have better dental appearance 48% attain straight teeth 3% that it was dentist recommendation 5% mentioned that they have been teased due to their dental irregularities 75% felt that their confidence and self-esteem would be increased if their teeth were straightened 64% stated that their social life would be improved 43% believed that their career opportunities would be brighter 20% improve dental health 20% enhance self confidence
